# Supplementary material for: Complexome profiling of the Chlamydomonas psb28 mutant reveals TEF5 as an early PSII assembly factor
Source: Plant Cell. 2025 Mar 17;37(6):koaf055. doi: 10.1093/plcell/koaf055 (PMC12164590; doi:10.1093/plcell/koaf055)
Supplement: koaf055_Supplementary_Data [file koaf055_supplementary_data.zip › psb28_interactive.zip!/plots/intensity/qualityPlots/peptides/ATPVD1_Cre03.g176250.t1.2.html]

### Cre03.g176250.t1.2

Trivial name: ATPVD1  
SeqCoverage [median]: 28.50  
Mass: 40.50 kDa  
  
 

| Sequence | MissedCleavage | Mass [Da] | wtIntensity | muIntensity | Score |
| --- | --- | --- | --- | --- | --- |
| ALNITLNSIGTELTRDDR | 1 | 2001.05 | 0.00 | 4870.44 | 50.39 |
| DGFLEGVVR | 0 | 990.51 | 967.18 | 5691.93 | 85.68 |
| IADGIVYLF | 0 | 1009.55 | 2707.96 | 0.00 | 94.16 |
| LGSTTAEIMGDMLAFETDRR | 1 | 2213.05 | 0.00 | 7516.85 | 71.70 |
| LGYGESQMLDK | 0 | 1239.58 | 4214.07 | 10015.66 | 155.57 |
| LLYEEEVKK | 1 | 1149.63 | 580.54 | 537.27 | 46.33 |
| LVNDWNYMR | 0 | 1209.56 | 0.00 | 762.42 | 72.55 |
| SAMEKVPAYSGIFSK | 1 | 1613.81 | 1575.36 | 13968.93 | 98.44 |
| VPAYSGIFSK | 0 | 1067.57 | 5336.73 | 12088.96 | 85.73 |
|  |  |  | 15381.8 | 55452.5 |  |
